# Supplementary material for: Improved Mechanical, Anti-UV Irradiation, and Imparted Luminescence Properties of Cyanate Ester Resin/Unzipped Multiwalled Carbon Nanotubes/Europium Nanocomposites
Source: Materials (Basel). 2021 Jul 29;14(15):4244. doi: 10.3390/ma14154244 (PMC8347775; doi:10.3390/ma14154244)
Supplement: Supplementary file 1 [file materials-14-04244-s001.zip › materials-1288891-supplementary.pdf]

*materials*

MDPI

*Article*

# Improved Mechanical, Anti-UV Irradiation, and Imparted Luminescence Properties of Cyanate Ester Resin/Unzipped Multiwalled Carbon Nanotubes/Europium Nanocomposites

**Na Yang [1], Xiaohua Qi [1], Di Yang [1], Mengyao Chen [1], Yao Wang [1], Linjun Huang [1], Olga Grygoryeva [2], Peter Strizhak [3], Alexander Fainleib [2] and Jianguo Tang [1,*]**

[1] Institute of Hybrid Materials, National Centre of International Joint Research for Hybrid Materials Technology, National Base of International Sci. & Tech. Cooperation on Hybrid Materials, Qingdao University, 308 Ningxia Road, Qingdao 266071, China; 17669490805@163.com (N.Y.); qixiaohua0930@163.com (X.Q.); yd130102@163.com (D.Y.); chenmengyao210@163.com (M.C.); wangyaoqdu@126.com (Y.W.); huanglinjun@qdu.edu.cn (L.H.)

[2] Institute of Macromolecular Chemistry of the National Academy of Sciences of Ukraine, 02068 Kyiv, Ukraine; grigoryevaolga@i.ua (O.G.); fainleib@i.ua (A.F.)

[3] B.L.V. Pysarzhevskii Institute of Physical Chemistry, National Academy of Sciences of Ukraine, 31 Prosp. Nauky, 03028 Kyiv, Ukraine; pstrizhak@hotmail.com

* Correspondence: tang@qdu.edu.cn

**Citation:** Yang, N.; Qi, X.; Yang, D.; Chen, M.; Wang, Y.; Huang, L.; Grygoryeva, O.; Strizhak, P.; Fainleib, A.; Tang, J. Improved Mechanical, Anti-UV Irradiation and Imparted Luminescence Properties of Cyanate Ester Resin/Unzipped Multiwalled Carbon Nanotubes/ Europium Nanocomposites. *Materials* **2021**, *14*, 4244. https://doi.org/10.3390/ma14154244

Academic Editor: Antonio Di Bartolomeo

Received: 22 June 2021
Accepted: 27 July 2021
Published: 29 July 2021

**Publisher's Note:** MDPI stays neutral with regard to jurisdictional claims in published maps and institutional affiliations.

**Copyright:** © 2021 by the author. Licensee MDPI, Basel, Switzerland. This article is an open access article distributed under the terms and conditions of the Creative Commons Attribution (CC BY) license (http://creativecommons.org/licenses/by/4.0/).

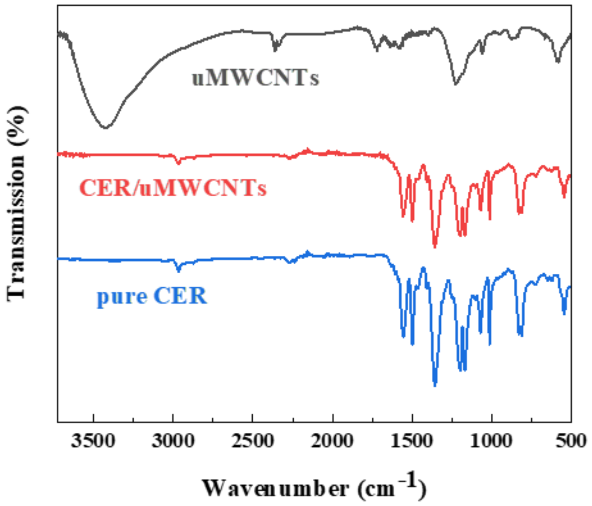


**Figure S1.** FTIR spectra of uMWCNTs, CER/uMWCNTs and pure CER.

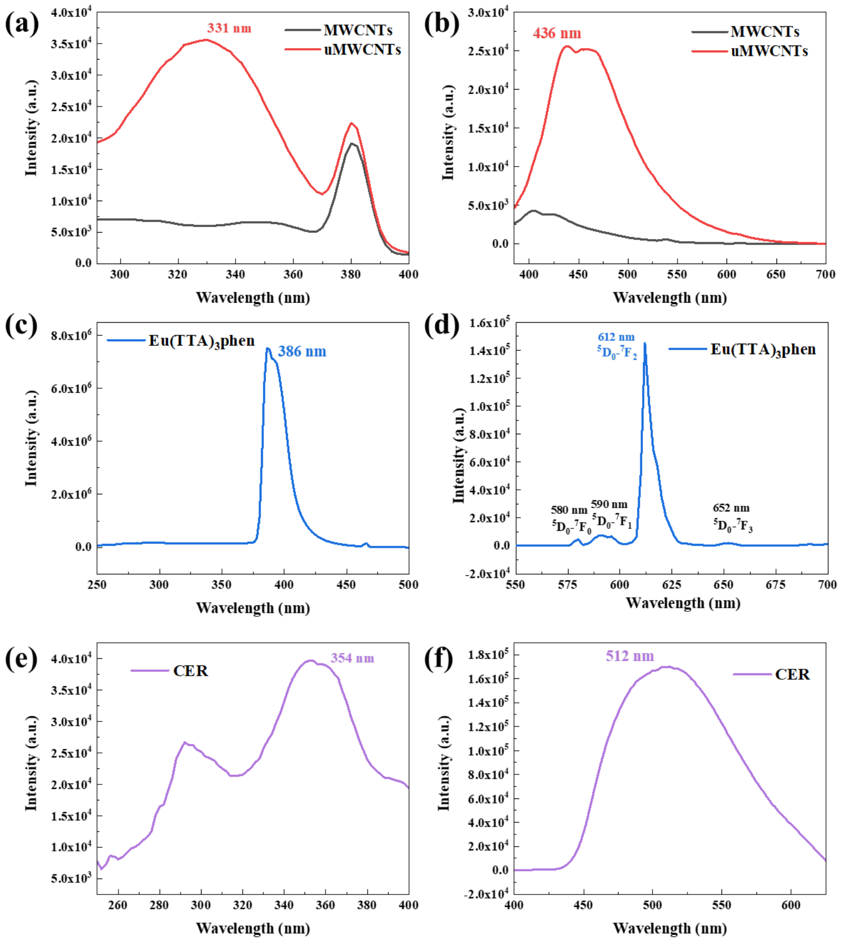


**Figure S2.** Luminescence excitation and emission spectra of MWCNTs and uMWCNTs (**a**,**b**), Eu(TTA)3phen (**c**,**d**) and CER (**e**,**f**).
